# Graduate medical education well-being directors in the United States: who are they, and what does the role entail?

**DOI:** 10.1186/s12909-024-05243-2

**Published:** 2024-03-08

**Authors:** Larissa R. Thomas, Jonathan A. Ripp, Jennifer G. Duncan

**Affiliations:** 1 Department of Medicine at Zuckerberg San Francisco General Hospital and Trauma Center, University of California, San Francisco School of Medicine, 1001 Potrero Ave 5H, 94110 San Francisco, USA; 2 Departments of Medicine, Medical Education, Geriatrics, and Palliative Medicine, Icahn School of Medicine at Mount Sinai, New York, USA; 3 Department of Pediatrics, Washington University in St. Louis School of Medicine, Missouri, USA

**Keywords:** Graduate medical education, Physician well-being, Burnout, Resident physicians, Physician wellness, Chief wellness officer, Academic leadership

## Abstract

**Background:**

Institutional Graduate Medical Education (GME) Well-being Director (WBD) roles have recently emerged in the United States to support resident and fellow well-being. However, with a standard position description lacking, the current scope and responsibilities of such roles is unknown. This study describes the scope of work, salary support, and opportunities for role definition for those holding institutional leadership positions for GME well-being.

**Methods:**

In November 2021, 43 members of a national network of GME WBDs in the United States were invited to complete a cross-sectional survey that included questions about job responsibilities, percent effort, and dedicated budget, and a free text response question about unique leadership challenges for GME WBDs. The survey was analyzed using descriptive statistics for quantitative data and thematic analysis for qualitative data.

**Results:**

26 members (60%) responded. Most were physicians, and the majority identified as female and White. Median percent effort salary support was 40%. A small minority reported overseeing an allocated budget. Most respondents worked to improve access to mental health services, oversaw institution-wide well-being programs, designed or delivered well-being content, provided consultations to individual programs, met with trainees, and partnered with diversity, equity, and inclusion (DEI) efforts. GME WBDs described unique challenges that had implications for perceived effectiveness related to resources, culture, institutional structure, and regulatory requirements in GME.

**Discussion:**

There was high concordance for several key responsibilities, which may represent a set of core priorities for this role. Other reported responsibilities may reflect institution-specific needs or opportunities for role definition. A wide scope of responsibilities, coupled with limited defined budgetary support described by many GME Well-being Directors, could limit effective role execution. Future efforts to better define the role, optimize organizational reporting structures and provide funding commensurate with the scope of work may allow the GME Well-being Director to more effectively develop and execute strategic interventions.

**Supplementary Information:**

The online version contains supplementary material available at 10.1186/s12909-024-05243-2.

## Background

With national calls to action in the United States to comprehensively address clinician well-being as largely a systems issue, institutions are building infrastructure to support organizational well-being beyond individual-level interventions [1–3]. In 2017, the Accreditation Council for Graduate Medical Education (ACGME) revised its Common Program Requirements (CPR) to address well-being in the clinical learning environment [4]. In alignment with national frameworks for addressing well-being [2, 5], these requirements not only direct a focus on efforts to improve individual resilience and mental health, but also emphasize the need to promote a culture of well-being while enhancing efficiency to maximize time for meaningful patient care.

The Chief Wellness Officer (CWO) role previously evolved in response to the need to integrate well-being work into C-Suite-level decision-making and strategic planning [6, 7]. Similarly, the emphasis on institutional responsibility to support resident and fellow well-being has led to a proliferation of institution-level positions, often termed “GME Well-being Directors” (WBD), who are dedicated to augmenting GME program director efforts to enhance resident and fellow well-being. While there are some broad parallels between GME Well-being Director and CWO roles, GME WBDs have unique challenges and responsibilities, in part because GME trainees and Offices of Graduate Medical Education are uniquely situated within institutional structures. Additionally, while residents and fellows have similar driver dimensions of well-being as other physicians as outlined by Shanafelt and Noseworthy [8], residents and fellows also face unique challenges that can exacerbate burnout. Stressors associated with the dual role of learner and worker during residency training, including long hours, lack of control/autonomy, competing priorities between work and education, and cognitive load, create significant workload demands. Additionally, social supports are important for residents who often move to a new location for training [9, 10]. Finally, residents and fellows are vulnerable to mistreatment due to their position within the hierarchy of the clinical system [11, 12].

To meet ACGME mandates to address these challenges and threats to GME well-being, GME WBD faculty roles have rapidly emerged without a “roadmap” to guide the scope of work and strategy for the role. The Collaborative for Healing and Renewal in Medicine (CHARM) GME Well-being Leaders Network (WLN), established in 2019, is a peer community that was developed to share best practices among those holding institutional GME well-being leadership positions. Initially, the GME WLN grew through informal networks to establish a learning community for individuals in this emerging role, then has continued to expand through outreach via professional organizations. As this community grew, the group members noted lack of defined job responsibilities for their newly emerging roles, leading to the desire for a member survey to elucidate this information. Additionally, member discussions noted that some dimensions of leadership challenges that they encountered were not fully captured in existing literature on well-being leadership strategy and the CWO role. Here, we report findings from a survey of this network related to GME WBD job responsibilities, describing current scope of work, salary support and perceptions of unique leadership challenges. Based on these findings, we also describe opportunities to optimize the role in order to allow the WBD to achieve maximal effectiveness as a strategic leader.

## Methods

As of October 2021, the CHARM GME WLN included 73 faculty and staff representing 57 institutions in the United States. We sent a cross-sectional, descriptive Qualtrics survey by email to 43 members within the GME WLN who held an institution-level GME well-being leadership position in November 2021. Those who had only department or program-level roles or who worked only with students, faculty, or other staff were excluded. Those who had broad institution-level well-being roles that were inclusive of GME were included. Questions were developed by the study leads based on literature describing the roles of the CWO [6], the ACGME Common Program Requirements for Well-being [4], and a review of prior discussion topics that had arisen in CHARM WLN group meetings. The timing of the survey coincided with group interest in defining evolution of roles post-pandemic. The final survey focused on job responsibilities, percent effort, and dedicated budget. The survey also included a free text question inquiring about leadership challenges for GME WBDs perceived as unique compared to those faced by well-being leaders responsible for other groups (for example, attending physicians or students). Several reminders were sent via email to eligible participants. Quantitative responses were analyzed using descriptive statistics. Two of the authors, who are also part of the GME WLN (JD and LT) used thematic analysis to group the qualitative responses. Themes were member-checked with the rest of the GME WLN at a meeting to verify resonance. This survey was deemed exempt by the UCSF Institutional Review Board.

## Results

### Who is the GME well-being director?

Twenty-six of 43 members with an institution-level GME well-being position responded (response rate 60%), representing 26 different institutions. Among respondents, 21 (81%) were female; 5 (19%) reported having a racial/ethnic identity other than white. Fourteen (54%) had an MD/DO degree, 7 (27%) had education degrees, and 5 (19%) had psychology, counseling, or social work degrees. Sixteen (62%) practiced clinically, and 10 (38%) were mental health professionals. Of 14 with an MD/DO degree, 11 practiced in medical-based specialties, 1 in a surgical-based specialty, and 2 in hospital-based specialties (ACGME defined groups [13]). Twenty (77%) worked at university-affiliated institutions, 2 (8%) at community-based institutions and 4 (15%) at hybrid or other institutions. Eleven (42%) had an institutional role solely dedicated to GME well-being, with no other well-being role or institution-level role in GME. For the remaining respondents, 6 (23%) had an institution-level GME role that included well-being as part of it but also included other responsibilities (e.g. DIO), 5 (19%) had an institution-level well-being role that included GME and other constituent groups (e.g. UME), and 4 (15%) had other roles such as co-director of mental health services. Most GME WBDs (85%) had funded roles, with percent salary support ranging from 5 to 100 (median 40%, mode 50%). A minority (4, 15%) reported having an unfunded institution-level role. Only 4 (15%) reported overseeing an allocated budget.

### GME well-being director responsibilities

For primary responsibilities, most respondents indicated working to improve access to mental health services, led institution-wide well-being programs, designed or directly delivered well-being content, provided consultations to individual program leadership, met with groups of trainees, and partnered with diversity, equity, and inclusion (DEI) efforts (Table 1). Most also indicated a reporting structure whereby they reported either to the Designated Institutional Official (DIO) or other institutional leaders. A majority also reported overseeing the institution’s approach to GME well-being, ensuring compliance with the ACGME CPRs for well-being, preparing for ACGME Clinical Learning Environment Review (CLER) visits [14], chairing a GME well-being committee, overseeing well-being surveys, and partnering with learning climate/mistreatment efforts. Less commonly, WBDs served on a medical staff well-being/impairment committee, oversaw mental health services, facilitated group reflection sessions, or led faculty development offerings. It was uncommon to provide direct individual or group mental health support, lead diversity or mistreatment efforts, or serve as a confidential ombudsperson for the institution.

**Table 1:** Responsibilities of GME Well-being Directors

Percentage of GME Well-being Directors	Role or responsibility
81–100	Improve access to mental health services Report to DIO or other institutional leaders Oversee wellness day or institution-wide well-being program Directly deliver well-being programming (educational modules, workshops, or lectures) for trainees Directly responsible for design and development of well-being programming or curricula Provide consultation to program directors or program coordinators Meet with groups of trainees to discuss well-being needs Partner with diversity, equity, and inclusion efforts
61–80	Oversee institutional GME well-being approach Ensure institutional compliance with GME common program requirements for well-being Prepare for CLER visits Chair GME well-being committee Oversee administration of well-being surveys Partner with learning climate/mistreatment efforts
41–60	Serve on medical staff well-being/impairment committee Oversee mental health services Conduct facilitated reflection sessions Lead formal faculty development offerings
21–40	Conduct group debriefing sessions Provide one-on-one mental health support for trainees Serve as ombuds or other confidential feedback mechanism for trainees
1–20	Lead diversity, equity and inclusion activities Lead learning climate/mistreatment efforts

### Perceived challenges in the role of GME well-being director

**Table 2:** GME Well-being Directors’ Perceptions of Unique Challenges in Their Roles

Resources	• Lack of funding/budget • Lack of space • Broad scope of role • Limited time for trainees to attend to well-being needs • Need for offerings to be at off-hours due to trainee schedules • Being asked to design, implement, and staff programming
Culture	• De-prioritization of well-being • Mismatch between institutional stated values and values in practice • Focus on individual well-being or social activities rather than systems and culture of well-being • “Underdog” or “us against them” trainee mentality • Hierarchy affecting reporting of mistreatment or toxic culture • Mixed reception by residents and fellows to institutional efforts, backlash against “wellness” initiatives • “It’s better now than it used to be,” rather than aspirational institutional mentality
Institutional Structure	• Lack of resident/fellow institutional memory diminishes impact of bigger changes that take time and decreases institutional impetus for investment • Resident and fellow needs can fall between the cracks for institutional accountability • Large number and diverse structure of programs • Lack of seat at table for systemic change • Lack of authority to mandate changes at institutional or program level • Disconnect among different institutional well-being leaders stemming from organizational structure • Making the case for alignment with other institutional efforts
Regulatory Requirements	• Tension between ACGME mandates for time to attend to well-being needs and health system status as workforce • Different specialty board/regulatory requirements can affect ability to make institution-specific policies supportive of well-being • Structural challenges of GME at national level can make it hard to improve structures locally (funding mechanisms separated from regulatory mechanisms)

GME Well-being Directors perceived several challenges as unique for those who lead work in GME well-being compared to those who lead well-being efforts for other groups. We grouped these perceived challenges into four broad themes: resources, culture, institutional structure, and regulatory requirements (Table 2). Resource challenges included issues related to budget, staff support, and space/time available for initiatives limited by tight trainee schedules. Cultural challenges centered around prioritization of well-being, a focus on individual well-being rather than workplace/systems issues, and hierarchy challenges. Challenges with institutional structure included lack of authority and influence of the GME WBD to create change, poor communication among institutional well-being leaders, and complex institutional (hospital vs. school of medicine) structures. Finally, regulatory challenges were reported with competing requirements between ACGME, specialty boards and institutional needs.

## Discussion

Among current GME WBDs, there is a high level of concordance for many responsibilities. Certain responsibilities, such as leading the strategic approach to well-being, establishing best practices, assessing trainee well-being, and consulting with training programs, could be considered a core set of responsibilities. Overall, the broad portfolio described by many WBDs, coupled with a lack of clearly defined budgetary support for the position, has potential implications for limiting the GME WBD’s effective role execution [15]. Less common responsibilities may relate to specific institutional needs and could represent opportunities for improved scope definition. For example, to allow GME WBDs to focus on strategic work, responsibilities such as in-depth content delivery to specific programs may best be delegated to individual program well-being leads who have commensurate support for this work. Furthermore, because the GME WBD is usually situated within the Office of Graduate Medical Education, it is also possible that roles and responsibilities that involve highly confidential or potentially disciplinary interactions with individual residents and fellows may introduce a conflict of interest that could negatively impact the director’s effectiveness as a system-level advocate. For this reason, some less-commonly held responsibilities, such as serving as an ombudsperson, providing direct mental health care, and serving on a medical staff well-being/impairment committee, perhaps should be delegated to other faculty or staff in institutions that have such capacity.

The low proportion of underrepresented racial/ethnic identities in our sample merits exploration of strategies to enhance diverse representation in this role and the need to partner with leaders in DEI. Additionally, as medical educators seek to train a diverse workforce representative of the broad population of patients they serve, attention to specific well-being needs of historically excluded groups to foster inclusion and belonging is also a pressing issue in GME [16]. While GME WBDs less commonly lead DEI or mistreatment efforts, most do indicate that they partner with DEI leaders and initiatives. Such partnerships can be a starting point for addressing well-being needs and impacts that may result from intersectional identities.

The unique challenges of working in GME are important to consider as roles and responsibilities are defined, and to determine the GME WBD’s optimal position in the organization to enable effective collaboration. Our GME WBD survey respondents outlined leadership challenges that they perceived were unique for their work, as compared to those tasked with leadership of well-being work for other populations such as students or faculty. While some of these challenges may also be faced by leaders in other domains outside of well-being (for example, those working in diversity, equity and inclusion, or those working in faculty development), unique aspects of these challenges are commonly encountered in graduate medical education work. Our respondents noted that these challenges were also often coupled with a lack of authority or institutional positioning to effect change. For example, the WBD may not have a seat at the table for important institutional decisions that affect trainee well-being, and rarely does the GME WBD have authority to enact or mandate a change at the institutional level. Additionally, the short time span spent in GME training can lead health systems to unconsciously or actively prioritize resources for longer-term employees over residents and fellows. Conversely, the turnover of trainees can also be protective against institutional complacency as new trainees advocate for changes to improve their experience.

Many of the GME WBDs had limited salary support and few had oversight of a budget. This finding may be related to historic underfunding of salary support for education roles, as well as limited independent budget support common in GME offices. Additionally, even when such positions are funded, the GME WBDs reported feeling asked to “do it all,” i.e., being tasked with design, implementation, and content delivery to programs. Being tasked with these responsibilities at the level of individual training programs leaves many GME WBDs feeling stretched in their capacity to oversee a strategic approach to developing effective interventions at the institutional level, particularly given the number and diversity of training programs. Such a task load for well-being leaders can make it challenging to design programming or interventions that meet the needs of a high proportion of the target group [7, 15].

Our paper describes typical current roles of the GME WBD, as well as challenges in role execution and deployment of effective interventions related to salary support, available program budget, the unique and time-limited nature of GME training, institutional structure, and regulatory requirements. Taken together, these findings can begin to suggest a consensus definition around this emerging role and ways to optimize the role for future success. We therefore outline below further considerations for next steps and an ideal future state for role definition, financial and administrative support, and institutional position within the organizational chart that would allow for effective partnerships for role execution.

First, to support scope definition, we propose an effort to clearly delineate whether the GME WBD is what we term an “actor*”* or an “advocate*”* for a given domain. "*"* responsibilities can be considered those that most naturally fall under the purview of the GME WBD, as opposed to other institutional leaders, and importantly, are also domains over which

**Table 3:** Potential Categorization of Actor vs. Advocate Responsibilities for GME Well-being Directors

Actor	Advocate
Institutional Well-being Expertise • Gather trainee feedback • Partner on internal reviews • Provide programmatic consultation • Give best practice guidance	Mental Health Supports • Share feedback on processes and access • Advocate for Needs • Advise on due process
Strategic Planning • Oversee GME-wide initiatives • Oversee strategy to meet ACGME compliance with well-being requirements • Chair well-being committee	Diversity, Equity, Inclusion • Partner with DEI offices and experts • Advocate and collaborate for intersectional needs related to well-being and DEI, mistreatment, and bias
Resource Collation and Dissemination • Mental health • Curricular resources • Faculty development • Crisis resources and support	Human Resources, Benefits, Campus Life, Facilities • Feedback and advocacy about benefits • Advocacy for programming • Advise about ACGME requirements (e.g., call rooms, lactation space) • Advocate for GME trainee needs
GME Well-being Assessment • Oversee institutional approach to GME well-being assessment • Review ACGME surveys • Disseminate data and opportunities to programs	System Supports • Advocate for GME-relevant EHR improvements and hardware needs • Partner with DIO and health system leads to advocate for improvements to the clinical learning environment

the GME WBD has *agency* to make changes within their own sphere of influence and chain of command. Actor responsibilities are the strategic work of the GME WBD. In contrast to actor roles, "advocate" roles are those for which the GME WBD can be expected to provide advice, feedback, or influence, but ultimately does not have authority, agency, or funding to implement a change. Informed by the survey responses, we propose a potential consideration for categorization of actor vs. advocate roles for the GME WBD in Table 3.

Next, GME WBDs need salary support and resources commensurate with their responsibilities, given their role in strategic planning and implementation [15]. There are many reasons why a particular institution may make the case to fund GME well-being work, and each well-being leader should consider their unique institutional needs and climate in considering how to “make the case” for additional institutional investment (7, 15–17). For example, the ACGME Section VI.C requirements [4] are a regulatory mandate that has spurred initial resource investment in many institutions, with subsequent investment justified in order to set aspirational goals. The so-called “moral imperative” to support well-being, the desire to support high quality patient care by supporting clinician well-being, and the “tragic case” imperative may be other motivating factors for institutions to fund well-being roles for GME trainees and other groups [15]. Our survey suggests that for most large training institutions, adequate salary support will need to be at least 20%, but may need to be as high as 40–50% depending on the scope of work and expected outcomes. Additionally, while independent control of a budget is not a requirement for this position, having some discretionary funding and administrative support may allow the WBD more autonomy in executing initiatives such as assessment tools, community-building activities, and mental health supports. While such recommendations apply to larger university-based institutions, smaller institutions may begin to initially resource this role by combining it with existing work; for example, by protecting a portion of someone’s time to work on well-being who is already funded to do “well-being-adjacent” work such as quality improvement or medical education (program director, associate program director, designated institutional official).

Finally, our findings support prior recommendations for institutional well-being leadership collaborations that cross disciplines and training levels. In an aspirational organizational structure, the GME WBD would ideally oversee or liaise with dedicated well-being leads within individual training programs or departments, while interfacing with both the institutional well-being leads (i.e., CWO) and DIO. Such partnerships would give the GME WBD a seat at the table to enhance role effectiveness, and could allow institutional alignment on strategy, messaging, and implementation for key well-being priorities across the organization [7, 15].

### **Limitations**

This study is a small cross-sectional sample of 26 individuals who were members of a learning community, and therefore is limited in its comprehensiveness. The majority of respondents were from university/academic institutions, and therefore this study may be less applicable to community institutions. Nevertheless, some were from community or hybrid institutions, and the data represent 26 different institutions across the US. The data presented here include early descriptive information about a newly emerging role. Since the time of this survey, this role has proliferated: the CHARM GME Well-being Leaders Network alone, which is not inclusive of all positions in the United States, now has more than 140 members, the majority of whom have roles as GME well-being directors (internal data). The continued expansion of this role since the time of this survey suggests that while some institutional leaders may remain hesitant to establish such roles, ongoing regulatory mandates, a sense of moral imperative, and a desire to support clinician well-being to ensure high quality patient care will likely continue to lead the growth of this role, regardless of direct evidence of a financial return on investment (7, 15–17). Based on challenges that GME well-being leaders continue to face, we believe that these results remain helpful not only for role definition and benchmarking information but also for initiating conversations about directions for future work.

## Conclusion

GME WBDs have an essential role in leading a strategic institutional approach to address unique well-being needs for residents and fellows. With the role initially emerging in response to regulatory mandates, it will benefit from further scope definition, funding commensurate with scope of work, and institutional support and buy-in to ensure role effectiveness in leading strategic work. Ultimately, the current national landscape and interaction of national and local policies and funding streams have direct impacts on the extent to which theWell-being Director can implement transformative change. Because local resource limitations can often lead to stagnation or de-prioritization of financial investment in systems interventions to improve GME well-being, national policies or guidelines with greater specificity and regulatory changes are ultimately necessary to expand resources that support systems change for GME trainees, faculty and staff.

### Electronic supplementary material

Below is the link to the electronic supplementary material.

Supplementary Material 1

## Data Availability

The datasets generated and/or analysed during the current study are not publicly available due to confidentiality reasons, but are available from the corresponding author on reasonable request.

## Abbreviations

ACGME: Accreditation Council for Graduate Medical Education
CHARM: Collaborative for Healing and Renewal in Medicine
CLER: Clinical Learning Environment Review
CPR: Common Program Requirements (for graduate medical education)
CWO: Chief Wellness Officer
DEI: Diversity, equity, and inclusion
DIO: Designated Institutional Official
GME: Graduate Medical Education
WBD: Well-being Director
WLC: Well-being Leaders Network
