# Supplementary material for: Graduate medical education well-being directors in the United States: who are they, and what does the role entail?
Source: BMC Med Educ. 2024 Mar 8;24:254. doi: 10.1186/s12909-024-05243-2 (PMC10921812; doi:10.1186/s12909-024-05243-2)
Supplement: Supplementary file 1 — Supplementary Material 1 [file 12909_2024_5243_MOESM1_ESM.pdf]

CHARM GME Well-being Leaders Network 2021 Survey

Start of Block:

Q1 Which of these statements best describes the scope of your role related to GME well-being?

- ○ I have an institutional-level GME role solely dedicated to well-being for trainees at my institution and no other institution-level GME responsibilities (1)
- ○ I have an institution-level GME role that includes well-being as part of it but also includes other responsibilities (e.g. DIO) (2)
- ○ I have an institution-level well-being role that includes GME and other constituent groups (e.g. UME) (3)
- ○ I am responsible for well-being for a subset of GME programs (4)
- ○ Other (please describe) (5) ____

Q2 What **percentage time** (full-time equivalent-FTE) do you receive for your institutional GME well-being role described above?

	0	10	20	30	40	50	60	70	80	90	100
Percentage time ()											

Q3 Which of the following elements are specific components of your GME well-being role?

	Yes (3)	No (4)	Unsure (5)
Ensure institutional compliance with GME CPR Section VI.C (1)	○	○	○
Serve on a Medical Staff physician well-being/impairment committee (2)	○	○	○
Prepare for CLER visits (3)	○	○	○
Improve access to mental health services (4)	○	○	○
Report to DIO or other institutional leaders (6)	○	○	○
Oversee wellness day or other institution-wide well-being program (7)	○	○	○
Chair GME Well-being Committee (8)	○	○	○
Oversee administration of well-being surveys (9)	○	○	○
Oversee institutional GME well-being approach (5)	○	○	○
Oversee mental health services (10)	○	○	○
Conduct group debriefing sessions (e.g. after adverse events) (11)	○	○	○
Conduct facilitated reflection sessions (e.g. for routine events) (12)	○	○	○

Provide one-on-one mental health support for trainees (13)	○	○	○
Serve as ombuds or other confidential feedback mechanism for trainees (14)	○	○	○
Meet with groups of trainees to discuss well-being needs (15)	○	○	○
Directly deliver well-being programming or curricula (educational modules, workshops, or lectures) for trainees (16)	○	○	○
Directly responsible for design and development of well-being programming or curricula (17)	○	○	○
Lead formal faculty development offerings (please describe) (18)	○	○	○
Provide consultation to program directors or program coordinators (19)	○	○	○
Responsible for leading DEI activities (describe) (20)	○	○	○
Partner with DEI efforts (describe) (21)	○	○	○
Responsible for leading learning climate/mistreatment efforts (22)	○	○	○
Partner with learning climate/mistreatment efforts (23)	○	○	○

Q5 What type of **funding** do you have to support the GME well-being activities that you oversee? (Check all that apply)

- ☐ No budget (1)
- ☐ Able to request funds for specific projects (2)
- ☐ Annual funding for specific programs only (e.g. wellness assessments, counseling services) (3)
- ☐ Approved budget that you oversee/control (please list amount) (4) ____
- ☐ Other (5) ____

End of Block:

Start of Block: Other Responsibilities

Q4 List other institutional committees or task forces you **LEAD** as a result of your GME well-being leadership role

____

____

____

____

____

Q6 List other institutional committees or task forces you are a **MEMBER** of as a result of your GME well-being leadership roles

____

____

____

____

____

Q7 List other administrative or leadership roles besides your GME well-being leadership role that **fund your salary**

____

____

____

____

____

End of Block: Other Responsibilities

Start of Block: Leadership Challenges

Q8 Please list up to 3 well-being challenges that are unique **for residents and fellows**, as opposed to other groups of learners or providers

○ Challenge 1 (1) ____

○ Challenge 2 (2) ____

○ Challenge 3 (3) ____

Q9 Please list up to 3 challenges that are unique **for well-being leaders in GME**, as opposed to well-being leaders for other groups of learners or clinicians

○ Challenge 1 (1) ____

○ Challenge 2 (2) ____

○ Challenge 3 (3) ____

End of Block: Leadership Challenges

Start of Block: Demographics

Q18 Which category best describes your training institution?

- ◯ University-based (1)
- ◯ Community-based (2)
- ◯ Other (specify) (3) ____

Q10 Do you practice clinical medicine currently?

- ◯ Yes (1)
- ◯ No (2)

Q11 Are you a mental health professional?

- ◯ Yes (1)
- ◯ No (2)

Q12 What is the area of your degree (select primary or terminal degree)?

- ◯ MD, DO, or equivalent physician degree (1)
- ◯ Psychology, social work, or counseling degree (specify) (2) ____
- ◯ Education degree (specify) (3) ____
- ◯ Other (specify) (4) ____

Display This Question:

If What is the area of your degree (select primary or terminal degree)? = MD, DO, or equivalent physician degree

Q13 Please select the category that best describes your **clinical specialty** (note: categories are per ACGME classifications)

Medical: Allergy/immunology, dermatology, family medicine, internal medicine and subspecialties, neurology, pediatrics and subspecialties, physical medicine and rehabilitation, psychiatry

Surgical: General surgery and subspecialties, neurosurgery, obstetrics/gynecology, ophthalmology, orthopaedic surgery, otolaryngology, plastic surgery

Hospital-based: Anesthesiology, emergency medicine, medical genetics, nuclear medicine, pathology and laboratory medicine, preventive medicine, radiology, radiation oncology,

- ○ Medical (1)
- ○ Surgical (2)
- ○ Hospital-based (3)

Q14 What is your current gender identity?

- ☐ Male (1)
- ☐ Female (2)
- ☐ Transgender male (3)
- ☐ Transgender female (4)
- ☐ Genderqueer/gender non-conforming (5)
- ☐ Additional category (please state) (6) ________________
- ☐ Prefer not to say (7)

Q15 How would you describe your race/ethnicity (select all that apply)?

- ☐ American Indian or Alaska Native (1)
- ☐ Asian (2)
- ☐ Black or African American (3)
- ☐ Hispanic or Latinx (4)
- ☐ Native Hawaiian or other Pacific islander (5)
- ☐ White or Caucasian (6)
- ☐ Additional category (please state) (7) ________________
- ☐ Prefer not to say (8)

Q16 Using your own definition of *burnout*, please select one of the answers below.

- ○ I enjoy my work. I have no symptoms of burnout. (1)
- ○ I am under stress, and don't always have as much energy as I did, but I don't feel burned out. (2)
- ○ I am definitely burning out and have one or more symptoms of burnout, e.g., emotional exhaustion. (3)
- ○ The symptoms of burnout that I am experiencing won't go away. I think about work frustrations a lot. (4)
- ○ I feel completely burned out. I am at the point where I may need to seek help. (5)

End of Block: Demographics
